# The War's Effect on a Special Hospital

**Published:** 1916-03-25

**Authors:** 


### THE WAR'S EFFECT ON A SPECIAL HOSPITAL.

### Phenomenal Increase in the Cost of Treatment.

As everybody knows, the resources of all hospitals have been seriously taxed by the advance in the price of drugs, but there are few institutions that have felt the strain due to this increase so seriously as the National Hospital for the Paralysed and Epileptic. It is true, of course, that some of the great general hospitals treating each year four or five times as many patients have been taxed more heavily; but in proportion to its size it is probably true to say that the resources of no hospital in the world have been so heavily drained as this one. This is due to the fact that the series of drugs which are most commonly prescribed in the treatment of nervous disease—namely, the bromides—have advanced in price to a phenomenal extent because the war has cut off the main source of supply of the raw material. While it has been possible to find suitable and less costly parallels for many of those products for the supply of which we were mainly dependent upon Germany before the war, unfortunately, no satisfactory substitute has been found for the salts of bromine. In a similar way other special hospitals have been affected. Thus all ophthalmic hospitals have suffered seriously from the shortage of such products as atropine and eserine, both of which are now worth more than their weight in gold; but when the total extra expenditure comes to be calculated it will not be found that the cost of drugs used in the treatment of eye diseases has been multiplied so many times as the cost of bromides.

A simple calculation will give some idea of how the increase in the price of bromides affects the drug bill at the Queen Square Hospital. Immediately before the war the makers' price of potassium bromide was about 1s. 6d. per lb.; there was a time, not so many years ago, when, as an outcome of a battle between the German and American makers, the price was run down to nearly 6d. per lb., but as that was a fictitious value, it will be better to take for the purpose of the calculation the pre-war figure of 1s. 6d. per lb. The immediate effect of the outbreak of hostilities was to lock up the supply of bromine in Germany, and the consequence was an upward movement in the price of bromides, which may not have stopped even now, when the value of potassium bromide is 25s. per lb.; and even at this enormous figure makers are obliged to exercise discretion in meeting the demands of their customers.

### An Increase of £3,948.

Incidentally it is interesting but disconcerting to observe that in Germany the abundant supplies of potassium bromide can be purchased at the rate of 8d. per lb.; so that German hospitals for the treatment of nervous disease are able to obtain this indispensable drug at about one-fortieth the price the National Hospital has to pay. But to proceed with our sum. The National Hospital uses in normal times a ton and a half of bromides a year. Assuming simply for the purpose of calculation that the whole of this is in the form of the potassium salt, it is clear that if the hospital had to buy this amount at the present market value the cost would be £4,200 sterling, or nearly seventeen times the cost at the pre-war rates. It will make the position clearer to set down the facts in the following way:—

Cost of 1½ Tons of Potassium Bromide.

| Before the War | In England to-day | In Germany to-day |
|---|---|---|
| £252 | £4,200 | £168 |

Fortunately, by judicious buying, the responsible authority at the institution in question has been able to discount this increase to a very considerable extent, but it is obvious that careless buying could easily have cost the hospital a substantial sum.

### Careful Buying and Careful Prescribing.

By systematically studying the condition of the market it has been possible to make purchases at progressive rates, and if the cost of the bromides that have been consumed in the hospital since the outbreak of the war, amounting approximately to 2¼ tons, were averaged out, it would be found that the cost per lb. has been something less than half the present price. But it is clear that the average price for the hospital's supplies during the second year of the war will not be far short of the present phenomenal figure. There is another way in which it has been found possible to effect some saving—namely, by giving preference, wherever possible, to the ammonium and sodium salts of bromine, both of which are less costly than the potassium salt. The reason for this difference in cost is that the world mainly depends for its supply of potash on the mineral deposits of Germany, so that all potassium salts are scarce, and while a pound of potassium bromide is worth 25s., the same quantity of ammonium bromide can be bought for 22s., and of sodium bromide for 18s. Of course, the hospital never did use potassium bromide exclusively, but as a result of an urgent request to the physicians to prescribe the sodium salt whenever this would serve the same purpose as the potassium salt a considerable economy has been effected; but in spite of all devices the drug bill of this hospital has vastly increased since the outbreak of the war; and so long as the war lasts the peculiar factor which has been responsible for this increase will continue to operate. The sum required to carry on the work of the hospital on a normal basis is about £18,000 a year, but it is clear that on the basis of the present price of bromides several thousand pounds must be added to this amount.

